# Epstein–Barr Virus and Human Herpesvirus-6 Reactivation in Acute COVID-19 Patients

**DOI:** 10.3390/v14091872

**Published:** 2022-08-25

**Authors:** Bailey Brooks, Christina Tancredi, Yufeng Song, Alemu Tekewe Mogus, Meei-Li W. Huang, Haiying Zhu, Tuan L. Phan, Harrison Zhu, Alexandra Kadl, Judith Woodfolk, Keith R. Jerome, Steven L. Zeichner

**Affiliations:** 1 College of Arts and Sciences, University of Virginia, Charlottesville, VA 22904, USA; 2 Department of Pediatrics, University of Virginia, Charlottesville, VA 22903, USA; 3 Department of Laboratory Medicine and Pathology, University of Washington, Seattle, WA 98195, USA; 4 Department of Internal Medicine, The Ohio State University Wexner Medical Center, Columbus, OH 43210, USA; 5 HHV-6 Foundation, Santa Barbara, CA 93108, USA; 6 School of Medicine, Baylor College of Medicine, Houston, TX 77030, USA; 7 Department of Medicine, University of Virginia, Charlottesville, VA 22903, USA; 8 Department of Pharmacology, University of Virginia, Charlottesville, VA 22903, USA; 9 Department of Microbiology, Immunology, and Cancer Biology, University of Virginia, Charlottesville, VA 22903, USA; 10 Vaccine and Infectious Disease Division, Fred Hutchinson Cancer Center, Seattle, WA 98109, USA

**Keywords:** COVID-19, SARS-CoV-2, Epstein–Barr virus, EBV, Human Herpesvirus-6, HHV-6, reactivation

## Abstract

Beyond their pulmonary disease, many COVID-19 patients experience a complex constellation of characteristics, including hyperinflammatory responses, autoimmune disorders, and coagulopathies. However, the pathogenesis of these aspects of COVID-19 is obscure. More than 90% of people are latently infected with the lymphotropic herpesviruses Epstein–Barr Virus (EBV) and/or Human Herpesvirus-6 (HHV-6). Some of the inflammatory features of COVID-19 resemble clinical syndromes seen during EBV and HHV-6 infection, and these latent viruses can be reactivated by inflammatory mediators. We hypothesized that EBV and HHV-6 reactivation might be a common feature of early COVID-19, particularly in patients with more inflammation. We tested for EBV and HHV-6 reactivation in 67 patients acutely hospitalized with COVID-19 using previously validated quantitative PCR assays on the plasma. In our cohort, we found that 15/67 (22.4%) patients had detectable EBV and 3/67 (4.5%) had detectable HHV-6. This frequency of activation is somewhat more than the frequency reported for some healthy cohorts, such as blood donors and other healthy control cohorts. There was no association between EBV or HHV-6 and markers indicative of more inflammatory disease. We conclude that EBV and HHV-6 activation at about day 7 of hospitalization occurred in a modest fraction of our cohort of COVID-19 patients and was not associated with high levels of inflammation. In the modest fraction of patients, EBV and HHV-6 reactivation could contribute to some features of acute disease and pre-disposition to post-acute sequelae in a subset of patients.

## 1. Introduction

Patients with COVID-19 exhibit a number of enigmatic clinical characteristics, such as hyperinflammatory responses, autoimmune phenomena, and coagulopathies, in addition to their pulmonary disease [1,2,3], but the etiology underlying these problems remains unknown. The hyperinflammatory responses, in turn, are thought to initiate additional pathophysiologic processes, such as rashes, vasculitis, coagulopathies, and myopathies. How these intertwined processes of SARS-CoV-2 infection and inflammation combine to yield the many, sometimes puzzling features is of clinical and scientific interest. Some features of COVID-19 resemble clinical diseases that accompany certain acute and chronic viral diseases, including diseases caused by lymphotropic herpesviruses.

A large majority of people have been infected with lymphotropic herpesviruses, including Epstein–Barr Virus (EBV), cytomegalovirus (CMV), and Human Herpesviruses-6 (HHV-6) and -7 (HHV-7) [4], which can remain latent within an individual’s cells throughout their lifetime. These viruses can emerge from latency when the infected host cells are triggered by various cues [5,6,7,8,9]. There is interest in studying EBV and/or HHV-6 activation in COVID-19 patients because, if EBV and/or HHV-6 reactivation are commonly observed and played a role in COVID-19 pathogenesis, the viruses might represent clinically useful therapeutic targets.

More than 90% of adults are infected with EBV [10]. EBV typically infects and then subsequently establishes a latent infection in the B lymphocytes, T lymphocytes, and some epithelial cells. EBV reactivation can occur when latently infected B cells differentiate into plasma cells, and this may involve the activation of cells via intracellular signaling pathways, including those acting downstream of the B-cell receptor and cytokine receptors [8,9,11,12,13,14,15,16,17].

Human Herpesvirus-6 (HHV-6) is the collective name for two distinct beta-herpesvirus species, HHV-6A and HHV-6B. Human Herpesvirus-6 (HHV-6), the causative agent of roseola [18], is a ubiquitous human pathogen [5,6,19,20] and consists of HHV-6A and HHV-6B, which share ~90% genetic identity but differ in their mode of cell entry [21]. Whereas HHV-6A primarily utilizes CD46, a ubiquitous complement regulatory protein, for cell entry, HHV-6B uses CD134, a molecule restricted to activated CD4+ T cells [21,22]. HHV-6 infects almost all children in the first few years of life. Infection is close to universal by the age of 3–4 years [23], and the seropositivity rate in adults in the U.S. is estimated at 95%.

HHV-6 infects a broad array of immune and non-immune cell types, including lymphocytes, monocytes, natural killer cells, dendritic cells, various neuronal cells, salivary gland cells, and epithelial cells [24]. HHV-6 can also integrate its entire genome into human DNA and can be inherited in a Mendelian fashion [25]. Similar to EBV, cytokines are believed to re-activate HHV-6. Its re-activation accompanies end-organ inflammatory processes in transplant patients and is also common in those undergoing cytotoxic chemotherapy [6,26]. Cytokines have also been implicated in HHV-6 reactivation associated with drug rash, with eosinophilia and systemic symptoms (DRESS syndrome) [27,28], and with drug-induced hypersensitivity syndrome [29,30].

Few studies have reported on the topic of viral re-activation in COVID-19 patients in early hospitalization. Some studies have examined EBV DNAemia in early COVID-19 and its association with the subsequent post-acute sequelae of SARS CoV-2 infection (PASC) [31], or later in PASC patients [32,33], but less is known about EBV and HHV-6 reactivations earlier in the disease course of COVID-19 patients. Patients with sepsis from several pathogens and patients in intensive care units generally can experience EBV activation, but it can be difficult to distinguish between the general effects of very severe illness and a response to a particular pathogen [34,35]. In the control groups for several studies investigating the associations of EBV and HHV-6 reactivation with a variety of diverse disorders, including fever without a source, fibromyalgia, multiple sclerosis, and cancer, almost all control participants without the disorder under consideration showed no evidence of viral DNA as a result of nucleic acid amplification [36,37,38,39,40,41,42,43,44], suggesting that a finding of herpesvirus DNAemia could be of interest in understanding COVID-19. In light of the very high prevalence of infection with EBV and HHV-6 in the general population, we hypothesized that hyperinflammatory processes in COVID-19 patients re-activate latent EBV and HHV-6, and that this is related to the inflammatory status of the patient. To address this hypothesis, we undertook a study of plasma obtained during hospitalization from a cohort of COVID-19 patients with severe disease. Our findings indicate that EBV and HHV-6 activation are not a common feature of acute COVID-19.

## 2. Materials and Methods

### 2.1. Clinical Cohort

The University of Virginia (UVA), Charlottesville, VA, USA, began to collect a prospective COVID-19 clinical sampling and natural history cohort. For this cohort, hospitalized patients diagnosed as having moderate to severe COVID-19 clinically by a confirmed nucleic acid amplification test were asked whether they would agree to be a participant in the study. Those agreeing signed UVA IRB-approved consent forms and were entered into the study. Clinical data were entered into the study database and samples were processed and stored in the UVA Biorepository and Tissue Facility.

The study participants were a subset of adults aged 18 years and above (*n* = 67). Following informed consent, the plasma samples were aliquoted and accessioned into the University of Virginia (UVA) Biorepository and Tissue Research Facility, and stored at −80 °C. We selected samples obtained on or about day 7 of hospitalization for this study, prior to the anti-inflammatory drug administration, in order to study patients early on in their hospital stay. The predominant viral strain for our sample was the alpha strain, with 98.51% of our sample (66 patients) being collected before July of 2021.The sample collection protocol was approved by the UVA IRB (HSR #200110), and approval was obtained to work on the specimens (HSR #HSR200362), following the Strengthening the Reporting of Observational Studies in Epidemiology (STROBE) guidelines for reporting results [45].

### 2.2. Patient Selection and Samples

For this study, we hypothesized that EBV and HHV-6 would exhibit reactivation in patients with more evidence of inflammation. Patient data were downloaded and subsequently processed from the study database and maintained in the UVA institutional REDCap database. In examining the laboratory tests available to characterize the patients’ inflammatory state, only two laboratory tests were available for a great majority of patients, the C-reactive protein and D-dimer. We therefore created a composite inflammatory score (CIS). We defined this CIS for each patient as the sum of the CRP value divided by the upper limit of normal for CRP plus the D-dimer value divided by the upper limit of normal for D-dimer:

CIS = CRP/ULN_CRP_ + D-dimer/ULN_D-dimer_

where “ULN” means “upper limit of normal”.

We calculated the CIS for the participants in the cohort and used the values for further analysis.

### 2.3. EBV and HHV-6 Assays

Patient plasma samples were tested for EBV and for HHV-6B and HHV-6A DNA using quantitative real-time polymerase chain reaction (qPCR) assays, as previously described [46].

### 2.4. Data Analysis

The Wilcoxon rank-sum test with continuity correction was used to determine whether there were associations between EBV and HHV-6 positivity and the clinical variables. The statistical analysis and data presentation were carried out in Rstudio with the included packages, stats and dplyr, and ggplot2 for the visualization, using the Rstudio environment. These rank-sum tests were conducted for EBV and HHV-6 positivity, and the clinical variables of the highest D-dimer level, highest CRP level, and inflammatory score were carried out using the wilcox.test function. Additionally, the correlation between the D-dimer and CRP levels was analyzed in Rstudio, graphically and numerically.

## 3. Results

We selected 67 patients for whom there were available samples for the assay and clinical data entered into the study databases, and for whom samples and at least D-dimer and C-reactive protein lab values were available. We assayed samples obtained at 7 d after hospitalization. Table 1 describes the selected demographic and clinical characteristics for the patients.

**Figure 1:**
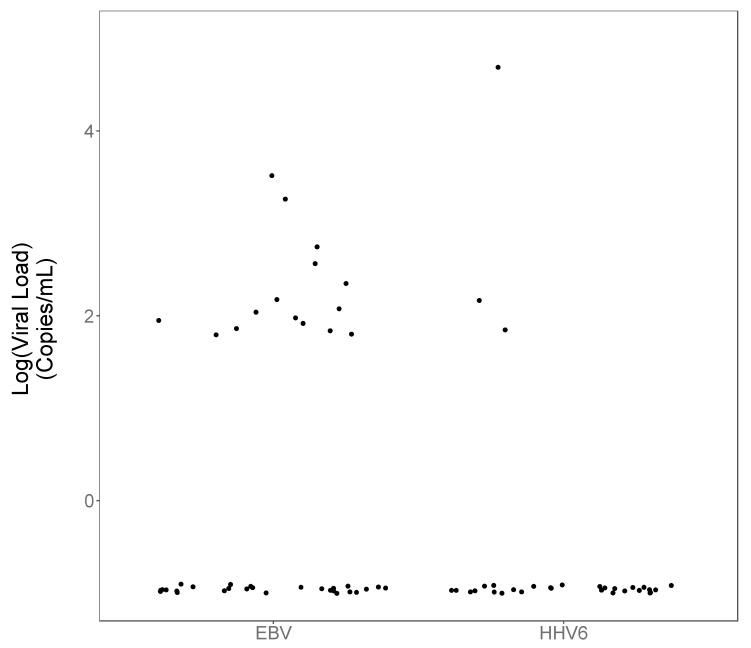
EBV and HHV-6 viral load values. The figure presents the viral load values (log_10_ copies/mL) for the patient samples studied. (An arbitrary 0.1 was added to all values prior to log transformation to obviate very large negative log values for results close to 0.).

Archived plasma samples from these participants were assayed for EBV and HHV-6 DNA by quantitative real-time PCR. A sample was assessed as positive for EBV or HHV-6 if the viral DNA was greater than 1 copy per reaction, which corresponds to a viral load of 50 copies/mL in the plasma. Of the 67 patient samples studied, 15 participants (22.4%) had detectable EBV DNA, and 3 participants (4.5%) had detectable HHV-6 DNA, with viral loads ranging from 2 to more than 4 log_10_ in the population that we studied. Figure 1 shows the viral load results for the 67 patients.

**Figure 2:**
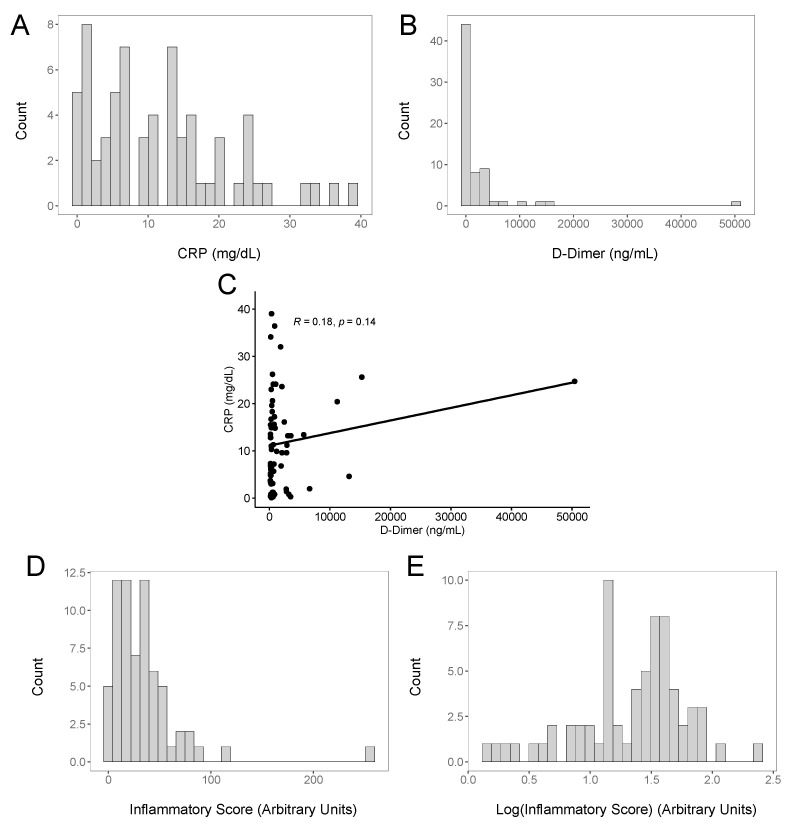
Inflammatory markers in acute COVID-19 patients. (**A**) Binned histogram of CRP values for the study population. (**B**) Binned histogram of D-Dimer values for the study population. (**C**) Correlation of CRP and D-Dimer values for each patient. (**D**) Binned histogram for the composite inflammatory score (CIS) for the study population. (**E**) Binned histogram of Log_10_-transformed CIS for the study population.

Since inflammation and pro-inflammatory cytokines are associated with herpesvirus reactivation, we used the clinical data available for the study patients to provide an assessment of their inflammatory state. Inflammatory cytokine levels were not routinely measured in our hospital, and only two markers that could help to evaluate the inflammatory state were obtained for a large fraction of the COVID-19 patients treated in our hospital: the C-reactive protein, a commonly used inflammatory marker [47,48], and the soluble fibrin degradation product indicative of ongoing coagulation and fibrinolysis, the D-Dimer [49,50]. Elevated D-Dimer levels indicative of a coagulopathy have been commonly observed in COVID-19 patients and have been associated with additional evidence of inflammatory processes [51] and with increased COVID-19 disease severity [52]. Figure 2 shows the distribution of the CRP (Figure 2A) and D-Dimer (Figure 2B) values for the patients in our study. To explore whether the CRP and/or D-Dimer levels tended to be elevated in the same patients, we plotted the CRP and D-Dimer values against each other (Figure 2C). While a few patients had high CRP and D-Dimer levels, the overall correlation was not strong, with an R-value of 0.18. For many COVID-19 patients with disease severe enough to require hospitalization in our cohort, elevations in the CRP or D-Dimer levels appeared to reflect different clinical manifestations. Because the CRP and D-Dimer values showed a poor correlation, we combined them into a composite inflammatory score (CIS), the sum of the CRP and D-Dimer values, normalized to the upper limit of normal for each assay (see Methods). We calculated the CIS values for each patient and plotted them (Figure 2D,E for the log_10_-transformed values).

**Figure 3:**
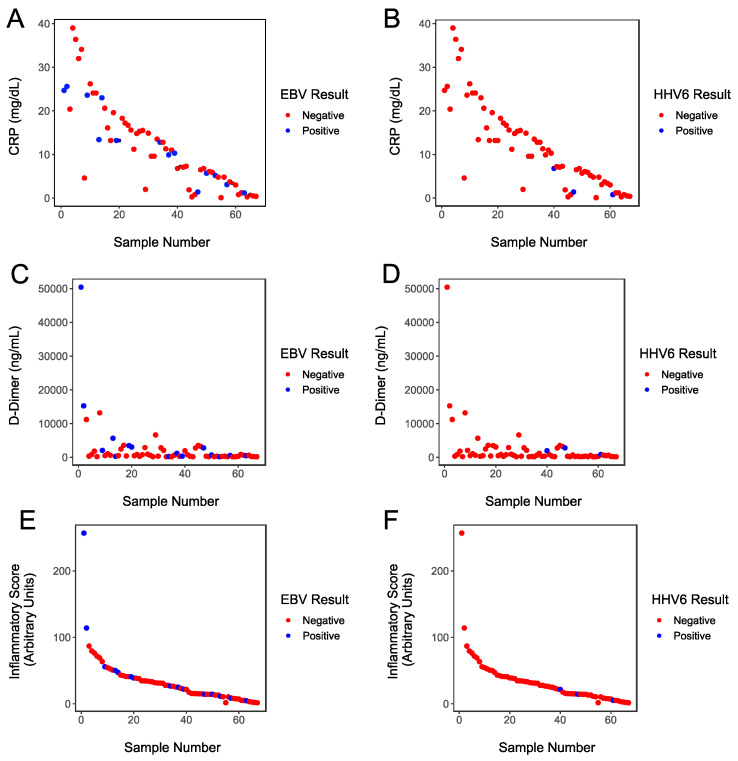
(**A**) CRP values plotted for each study participant. Values for patients with negative EBV assays are indicated in red and values for patients with positive EBV assays are indicated in blue. (**B**) CRP values plotted for each study participant. Values for patients with negative HHV-6 assays are indicated in red and values for patients with positive HHV-6 assays are indicated in blue. (**C**) D-Dimer values plotted for each study participant. Values for patients with negative EBV assays are indicated in red and values for patients with positive EBV assays are indicated in blue. (**D**) D-Dimer values plotted for each study participant. Values for patients with negative HHV-6 assays are indicated in red and values for patients with positive HHV-6 assays are indicated in blue. (**E**) CIS values plotted for each study participant. Values for patients with negative EBV assays are indicated in red and values for patients with positive EBV assays are indicated in blue. (**F**) CIS values plotted for each study participant. Values for patients with negative HHV-6 assays are indicated in red and values for patients with positive HHV-6 assays are indicated in blue. For visualization purposes, to highlight any possible associations between inflammatory state and virus reactivation, the samples were first ranked by CIS prior to drawing the figures.

To explore the hypothesis that a hyper-inflammatory state is more likely to be associated with EBV and HHV-6 activation, we examined the relationship between patients who had viral reactivation and those patients’ inflammatory marker values, arranging the patients’ data by ranking the patients by CIS. Figure 3A shows the relationship between CRP and EBV positivity, Figure 3B shows the relationship between CRP and HHV-6 positivity, Figure 3C shows the relationship between D-Dimer and EBV positivity, Figure 3D shows the relationship between D-Dimer and HHV-6 positivity, Figure 3E shows the relationship between CIS and EBV positivity, and Figure 3F shows the relationship between CIS and HHV-6 positivity. The data were examined to determine whether there were associations between EBV and HHV-6 positivity status and CRP, D-Dimer, or CIS, and no statistically significant associations were identified.

## 4. Discussion and Conclusions

Our study examined the hypothesis that the reactivation of latent EBV and HHV-6 commonly occurs in patients with moderate to severe acute COVID-19. We observed EBV and HHV-6 reactivation at the time of testing, but only in a fraction of the patients that we studied. This reactivation may have been related to COVID-19 or other factors. Nevertheless, we did not observe extensive EBV and HHV-6 reactivation, which we had hypothesized that we would observe.

Our study had several strengths. Specifically, the tested samples were collected prospectively from acutely hospitalized patients with a well-established diagnosis of COVID-19 relatively early in the course of disease. Additionally, the number of patients studied was large enough to ensure that, were EBV or HHV-6 reactivation to occur in a large fraction of the acutely ill patients in our cohort, we would have likely detected the reactivation. Furthermore, the activation of EBV and HHV-6 was determined using highly sensitive and specific assays that have previously been used to characterize other cohorts.

Nonetheless, our study had several limitations. We did not study all the available patients, but only a subset who had available data for the inflammatory marker assays. Although unlikely, it is possible that this selection biased our choice of samples for the study. It would have been ideal to have a contemporaneously collected set of samples from control patients without COVID-19, but since this study was conducted using banked samples from a previously established natural history cohort study, this was not possible. We assayed for EBV and HHV-6 activation at a single time point, about a week after the patients were hospitalized. Thus, the data do not address potential EBV and HHV-6 activation during the very early stages of COVID-19 infection or at later time points, including in patients with post-acute sequelae of the SARS-CoV-2 infection. In a study of EBV activation in acute sepsis patients, the rate of activation increased over the course of the hospitalization; thus, activation occurring at one time point, followed by a disappearance of the reactivation, would be unlikely [34]. For HHV-6, in Drug Rash with Eosinophilia and Systemic Symptoms (DRESS) syndrome, HHV-6 reactivation can be delayed until 2–4 weeks after the syndrome onset, but then it can continue for a long time [53]. In addition, no patients with MIS-C were tested. The data do not address EBV and HHV-6 activation in the larger fraction of COVID-19 patients who were not admitted to the hospital. There may be some particular patient characteristics that are associated with EBV activation in COVID-19 patients but were not identified in this study that may require future work. We also only studied patients who had samples available around day 7 of hospitalization, and so patients who had been discharged, transferred, or died prior to day 7 of hospitalization were not studied.

The frequency of EBV DNAemia in normal study participants varies considerably from one report to another. For example, a study of 508 healthy Portuguese blood donors found that 37.2% had detectable EBV DNA in their peripheral blood samples [54]; a survey of 673 healthy blood donors of various original nationalities residing in Qatar found that 52.6% had detectable EBV in their peripheral blood [55]; and a survey of 100 healthy blood donors in Southeast Texas commonly observed detectable EBV viremia in 72% of the donors [56]. However, in contrast to those generally high levels of EBV reactivation in healthy blood donors, in a study of the reactivation of multiple viruses in septic patients, among 165 older normal controls (60 years mean, range 60–72), who were matched to the septic patients, only 3.6% of the healthy controls had EBV detected in their blood samples, while EBV was detected in 53.2% of the septic patients [34]. In a study of COVID-19 patients that aimed to identify possible predictors of post-acute sequelae of COVID-19 (PASC), EBV activation was detected in 14% of 209 COVID-19 patients at the initial time of observation [31]. In this study, for the patients with detectable EBV, the viral loads dropped 2–3 fold for samples obtained at entry into the study compared to a sample obtained later during the acute phase of the illness. The rate of EBV reactivation that we observed in our cohort of acutely hospitalized COIVD-19 patients (22.4%) was moderately higher, but it was within the range reported for some of the normal cohorts described above.

The reasons for the differences in these rates of observed EBV reactivation in different healthy populations have not been clearly determined. There have been associations observed between EBV reactivation and COVID-19-associated disorders. Notably, an association has been observed between EBV viremia, as one of four risk factors for post-acute sequelae of PASC, with type-2 diabetes, SARS-CoV-2 viremia, and certain autoantibodies [31]. The overall rate of EBV DNAemia observed in that study—14%—was similar to the 20% rate we observed and the rates reported in healthy blood donors, as described above, suggesting a rate of reactivation in COVID-19 patients similar to that observed in the general population, but that such an activation may be associated in some way with PASC, or that other factors predispose both to EBV DNAemia and PASC.

Some reports, mostly from China, and mostly describing findings in patients infected with the original Wuhan strain, described EBV findings in relation to COVID-19. One study reported higher CRP levels in patients with higher EBV antibodies against viral capsid antigen (VCA) [57]. EBV DNAemia has been reported in COVID-19 patients hospitalized in the intensive care unit at levels significantly greater than in patients in the same hospital who were not hospitalized in the intensive care unit [58]. In a study of COVID-19 patients, those with positive EBV serology appeared to have significantly better outcomes if they were treated with ganciclovir [59].

Studies have observed an increased, but not universal, EBV reactivation in PASC patients [32,33]. While EBV and HHV-6 activation are undoubtedly important in many disease states, our findings suggest that the reactivation of these viruses was not a very common feature among the COVID-19 patients evaluated in our study after about 7 days of hospitalization.

## Figures and Tables

**Table 1:** Patient Demographic and Clinical Characteristics (IQR: interquartile range).

Characteristic	*n* = 67, Median, (IQR)
*Age at Admission*	60 (48, 66)
*First Weight (kg)*	91 (76, 112)
Unknown	1
*Height (m)*	1.68 (1.60, 1.83)
Unknown	6
*Gender*	
Female	28 (42%)
Male	39 (58%)
*Race*	
African American	23 (34%)
Native American	1 (1.5%)
Caucasian	31 (46%)
Other	12 (18%)
*Smoking Status*	
Current	1 (2.1%)
Former	14 (29%)
Never	24 (50%)
Unknown	9 (19%)
NA	19
*Need for ICU*	
No	28 (42%)
Yes	39 (58%)
*Need for Intubation*	
No	41 (61%)
Yes	26 (39%)
*Blood Type*	
A−	2 (4.8%)
A+	14 (33%)
AB+	1 (2.4%)
B+	3 (7.1%)
O−	1 (2.4%)
O+	21 (50%)
Not determined	25
*Highest Temperature*	
<38	42 (49%)
101	12 (18%)
>38.5	12 (18%)
>40	1 (1.5%)
*Length of Stay (Days)*	10 (5, 22)
*Time to Death (Days)*	
6	1
10–20	2
20–30	3
>30	2
*Highest D-Dimer*	605 (280, 2074)
*Highest CRP*	10 (4, 16)
*BMI*	33 (27, 38)
*Need for Vasopressors*	
Yes	44 (66%)
*Dexamethasone*	
Yes	41 (61%)
*Prednisone*	
Yes	3 (13%)
*Methylprednisolone*	
Yes	5 (22%)
*Tocilizumab*	
Yes	4 (6%)
*Remdesivir*	
Yes	27 (40%)
*Creatinine*	1.00 (0.80, 1.45)
*Hgb*	12.4 (10.8, 13.6)
*IFNa3*	45 (36, 72)
Not determined	45
*Highest Ferritin*	1277 (7, 7161)
Not determined	5
*Highest Troponin*	
<0.04	47 (73%)
0.04–0.4	12 (18%)
>0.6	3 (5%)
Not determined	5
*Highest Creatine*	1.10 (0.90, 1.80)

## Data Availability

Data are presented in the manuscript. Raw data can be obtained on request.
